# Dietary Habits and Their Impact on Gastroesophageal Reflux Disease (GERD)

**DOI:** 10.7759/cureus.65552

**Published:** 2024-07-27

**Authors:** Muzamil Khan, Kinjal Shah, Satkarjeet Kaur Gill, Nida Gul, Jestin K J, Vivian Valladares, Laiba Ali Khan, Muhammad Raza

**Affiliations:** 1 Internal Medicine, George Washington University School of Medicine and Health Sciences, Washington, DC, USA; 2 Health Administration, Edward J. Bloustein School of Planning and Public Policy, New Jersey, USA; 3 Internal Medicine, Jagare Ridge Medical Clinic, Edmonton, CAN; 4 Medicine, MTI Khyber Teaching Hospital Peshawar, Peshawar, PAK; 5 Department of Pathology and Laboratory Medicine, All India Institute of Medical Sciences, Bilaspur, Bilaspur, IND; 6 Medicine, Universidad Iberoamericana, Miami, USA; 7 Internal Medicine, MTI Khyber Teaching Hospital Peshawar, Peshawar, PAK

**Keywords:** diet, eating habits, dietary habits, lifestyle, gastroesophageal reflux disease

## Abstract

Introduction

Gastroesophageal reflux disease (GERD) is marked by the frequent occurrence of stomach acid flowing back into the esophagus, causing symptoms such as heartburn and acid regurgitation at least once a week. When reflux leads to troublesome symptoms and esophageal damage and adversely affects quality of life, it is diagnosed as GERD. Age, gender, ethnicity, genetic predispositions, and aspects of diet and lifestyle, including factors like obesity and smoking, are associated with GERD.

Methodology

This cross-sectional study was conducted within the Departments of General Medicine, Surgery, and Gastroenterology at Khyber Teaching Hospital (KTH) in Peshawar, spanning from January 2024 to June 2024. Patients who visited these departments or the Outpatient Department within the specified period with GERD were included in the study. A non-probability purposive sampling technique was used. For the analysis, we utilized IBM SPSS Statistics version 21.

Results

This study consists of 280 participants. The mean age of the participants in this study was 44.60 years. GERD has a significant association with obesity (69.99) and lack of exercise (80%), and a negative association was found between other gastrointestinal conditions (55.71%) and smoking (64.28). Common symptoms among GERD patients were swallowing difficulty, regurgitation, heartburn, and chest pain.

Conclusion

Our study is the first to examine the relationship between lifestyle factors and GERD among Pakistani patients. Our findings highlight significant associations between GERD and several factors, including gender, BMI, dietary habits, and lack of exercise. Notably, cultural and regional differences appear to influence GERD prevalence and its risk factors, as demonstrated by the minimal impact of alcohol consumption in our study population.

## Introduction

Gastroesophageal reflux disease (GERD) is marked by the frequent occurrence of stomach acid flowing back into the esophagus, causing symptoms such as heartburn and acid regurgitation at least once a week [1]. In Western countries, where GERD is most common, the prevalence rates have been documented to be between 10% and 20% [2]. Concurrently, the evolution toward modern living standards, along with associated lifestyle shifts and a faster pace of life, has contributed to a rising incidence of symptomatic GERD in China, reaching 3.8% in 2016 [3]. The worldwide prevalence of GERD has been documented at 13.98%. It is more commonly observed at higher rates (10.0-33.0%) in North America, Europe, and the Middle East, varying between 2.5% and 7.8% in East Asia, and aligning closely with Western countries (10.0-20.0%) in Turkey [4].

Gastroesophageal reflux (GER) is characterized by the involuntary movement of stomach contents into the esophagus, a process that normally occurs during the stomach's adjustment to food after meals, even in healthy individuals. This reflux can occur due to temporary relaxation of the lower esophageal sphincter (LES) or the inability of the sphincter to adjust to changes in intra-abdominal pressure. The stomach contents that reflux into the esophagus typically include acid, pepsin, and occasionally bile. Physiological reflux is brief and typically asymptomatic. However, when reflux leads to troublesome symptoms and esophageal damage and adversely affects quality of life, it is diagnosed as GERD [5-6].

Age, gender, ethnicity, genetic predispositions, and aspects of diet and lifestyle, including factors like obesity and smoking, are associated with GERD [7,8]. Treatment typically involves the use of proton pump inhibitors (PPIs), medications known for their efficacy. However, prolonged PPI use has been linked to increased risks of bone fractures, chronic or acute kidney disease, pneumonia, and gastrointestinal infections [5].

A notable percentage of patients, estimated between 10% and 40%, do not experience sufficient relief from PPI treatment. This condition, termed refractory GERD (rGERD), not only diminishes patients' quality of life but also substantially escalates healthcare expenses. Untreated, the condition can progress to complications such as esophageal narrowing (stricture), gastrointestinal bleeding, and esophageal adenocarcinoma [9].

The rationale for our study is to explore the relationship between dietary habits and the prevalence of GERD in Pakistan. By examining dietary patterns and their potential association with GERD, we aim to better understand the dietary factors contributing to this condition within our local population. This research is crucial for developing targeted dietary recommendations and interventions to mitigate the impact of GERD and improve public health outcomes in our community.

## Materials and methods

This cross-sectional study was conducted within the Departments of General Medicine, Surgery, and Gastroenterology at Khyber Teaching Hospital (KTH) in Peshawar, spanning from January 2024 to June 2024. Approval for data collection was granted by the Medical Director of KTH and the Director of Medical Education at Khyber Medical College (approval no. 284/DME/KMC), ensuring compliance with ethical standards.

Participants were selected based on their presentation with typical symptoms of GERD, such as regurgitation, nausea, and heartburn, and those who were diagnosed with GERD either clinically or through esophagoscopy or 24-hour pH monitoring. Patients who visited these departments or the Outpatient Department within the specified period were included in the study. A purposive sampling method, which is non-probability-based, was employed.

All patients admitted to the gastro unit for reasons other than GERD and those who were not willing to take part in the study were excluded. Consent was obtained from all participants before their inclusion in the study. Questionnaires were filled by participants using the interview method (for the survey questionnaire, see Appendix).

Data analysis

For the analysis, we utilized IBM SPSS Statistics for Windows, Version 21.0 (released 2012, IBM Corp., Armonk, NY). The body mass index (BMI) of each participant was calculated by dividing their weight in kilograms by the square of their height in meters. Participants with a BMI exceeding 30 were categorized as obese. To determine the association between GERD and its various risk factors, we thoroughly examined the symptoms and factors commonly found in the majority of GERD patients. Due to the nature of our sample, which included only GERD-positive cases, we were unable to apply the chi-square test. Consequently, we adopted this method to establish associations. The results were presented using tables and bar charts for clear visualization of the data.

This methodological approach ensured a comprehensive analysis of the correlation between dietary habits, obesity, and the prevalence of GERD among the study population, providing valuable insights for future research and clinical practice.

## Results

This study consists of 280 participants. The mean age of the participants in this study was 44.60 years with a standard deviation of 12.76 years. A chi-square test was conducted to examine the association between GERD and gender. The results indicated that GERD is significantly more prevalent in females having 164 positive cases in females, suggesting a strong association between gender and GERD. Details are given in Table 1.

**Table 1:** Age of the participants

Age of the participants	Male	Female	Total n (%)
<30 years	16	16	32 (11.2)
30-50 years	96	100	196 (70)
>50 years	4	48	52 (18.57%)

The study found that GERD is more common in overweight patients, with 69.99% of patients having a BMI greater than normal, indicating a strong association between higher BMI and the prevalence of GERD.

The study did not identify any association between GERD and other gastrointestinal conditions, such as inflammatory bowel disease and Crohn's disease, with 55.71% of patients having GERD not having these chronic gastrointestinal conditions. In addition, an increased frequency of meals was also significantly associated with GERD, with 88.57% of patients who ate three or greater than three meals per day. These findings suggest that individuals who eat more frequently are more likely to develop GERD.

The study did not find any significant association between GERD and smoking as 64.28% of patients having GERD were non-smokers, and the study found out association between GERD and not doing exercise as 80% of GERD patients were not doing exercise. This suggests that while physical activity levels influence the prevalence of GERD in the studied population, smoking habits do not.

Details are given in Table 2.

**Table 2:** Factors affecting gastroesophageal reflux disease (GERD)

Variables		Male	Female	Total n (%)
BMI of the participant	18.5-24.9	32	52	84 (30)
	25-29.9	52	92	144 (51.42)
	>30	32	20	52 (18.57)
For how long you have been diagnosed with GERD?	<1 year	60	16	76 (27.14)
	1-2 years	24	36	60 (21.42)
	>2 years	32	112	144 (51.42)
Do you have any other gastrointestinal conditions (e.g., irritable bowel syndrome (IBS), Crohn’s disease)?	Yes	64	60	124 (44.28)
	No	52	104	156 (55.71)
How many meals do you eat per day?	One	0	12	12 (4.28)
	Two	0	20	20 (7.14)
	Three	84	80	164 (58.71)
	More than three	32	52	84 (30)
Do you smoke?	Yes	100	0	100 (35.71)
	No	16	164	180 (64.28)
Do you exercise regularly?	Yes	56	0	56 (20)
	No	60	164	224 (80)
Do your symptoms affect your daily activities?	Yes	56	20	76 (27.14)
	No	60	144	204 (72.85)

The participants demonstrated a variety of eating habits that influence GERD symptoms. Many participants occasionally consumed fatty or fried foods, which can stimulate the production of gastric juice and exacerbate GERD symptoms by increasing the volume of acid available to reflux into the esophagus.

Fatty foods slow down gastric emptying, prolonging the time food remains in the stomach and thereby increasing the potential for reflux.

Citrus foods and juices, which are highly acidic, were rarely consumed by the participants. This lower frequency may help reduce acid reflux episodes since these foods can increase stomach acidity. Tomato-based products, also acidic, were consumed occasionally, which might sometimes contribute to GERD symptoms.

Caffeinated drinks were consumed occasionally. Caffeine can relax the LES and increase stomach acid, potentially worsening GERD symptoms. Carbonated drinks, which can cause bloating and increase pressure on the LES, were rarely consumed by participants. Eating large meals, which can expand the stomach and increase pressure on the LES, was also a rare occurrence. Occasionally, participants ate within two hours before bedtime, a habit that can exacerbate GERD symptoms at night by making it easier for stomach acid to enter the esophagus when lying down. Lastly, chocolate, which can relax the LES and increase acid production, was mostly never consumed by participants.

**Figure 1:**
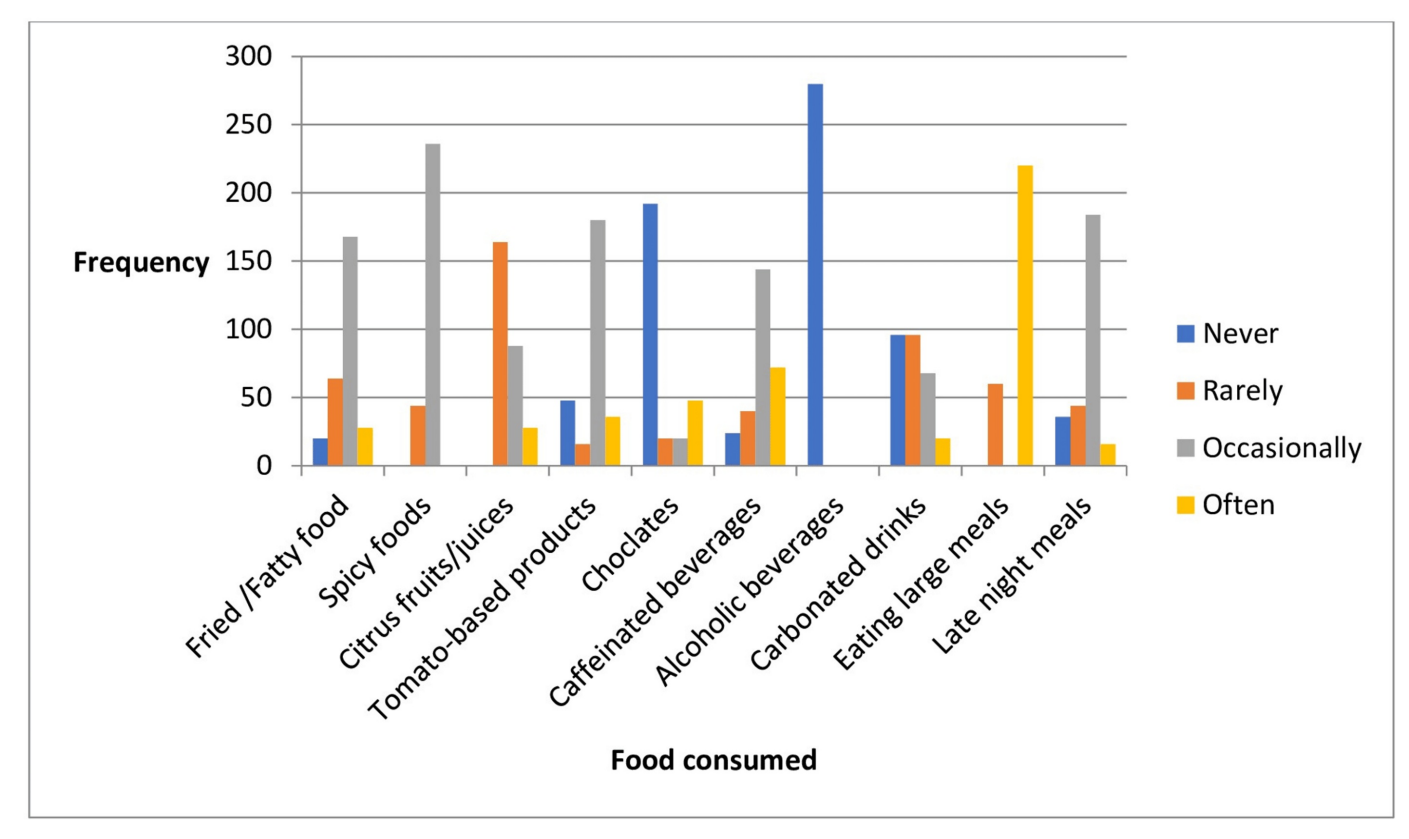
Eating habits of gastroesophageal reflux disease (GERD) patients

Overall, the participants tended to avoid or limit foods and behaviors that are known to trigger GERD symptoms, which may help in managing their condition effectively. Details are given in Table 3 and Figure 1.

**Table 3:** Eating habits of gastroesophageal reflux disease (GERD) patients

Eating habits		Male	Female	Total n (%)
How often do you consume fried or fatty food?	Never	0	20	20 (7.14)
	Rarely	16	48	64 (22.85)
	Occasionally	100	68	168 (60)
	Often	0	28	28 (10)
How often do you consume spicy food?	Never	0	0	0 (0)
	Rarely	44	0	44 (15.71)
	Occasionally	72	164	236 (84.28)
	Often	0	0	0 (0)
How often do you consume citrus fruits/juices?	Never	0	0	0 (0)
	Rarely	116	48	164 (88.57)
	Occasionally	0	88	88 (31.42)
	Often	0	28	28 (10)
How often do you consume tomato-based products?	Never	0	48	48 (17.14)
	Rarely	16	0	16 (5.71)
	Occasionally	100	80	180 (64.28)
	Often	0	36	36 (12.85)
How often do you consume chocolates?	Never	32	160	192 (68.57)
	Rarely	16	4	20 (7.14)
	Occasionally	20	0	20 (7.14)
	Often	48	0	48 (17.14)
How often do you consume caffeinated beverages (coffee, tea, soda)?	Never	0	24	24 (8.57)
	Rarely	0	40	40 (14.28)
	Occasionally	116	144	144 (51.42)
	Often	0	72	72 (25.71)
How often do you consume alcoholic beverages?	Never	116	164	280 (100)
	Rarely	0	0	0 (0)
	Occasionally	0	0	0 (0)
	Often	0	0	0 (0)
How often do you consume carbonated drinks?	Never	0	96	96 (33.21)
	Rarely	48	48	96 (33.21)
	Occasionally	68	0	68 (24.28)
	Often	0	20	20 (7.14)
How often do you eat large meals?	Never	0	0	0 (0)
	Rarely	60	0	60 (21.42)
	Occasionally	0	0	0 (0)
	Often	56	164	220 (78.57)
Do you eat at late night (within two hours of going to bed)?	Never	0	36	36 (12.85)
	Rarely	4	40	44 (15.71)
	Occasionally	96	88	184 (65.71)
	Often	16	0	16 (5.71)

The participants in the study experienced various GERD symptoms with differing frequencies. Heartburn was reported often, indicating it as a common symptom among the participants. Regurgitation and chest pain were experienced occasionally, suggesting that these symptoms were less frequent but still notable. Difficulty in swallowing was almost never reported, indicating it was a rare symptom among the participants. These findings highlight the prevalence and variability of GERD symptoms within the study group. Details are given in Table 4.

**Table 4:** Symptoms experienced by the gastroesophageal reflux disease (GERD) patients

Symptoms		Male	Female	Total n (%)
How often do you experience heart burn?	Never	0	0	0 (0)
	Rarely	44	12	56 (20)
	Occasionally	40	72	112 (40)
	Often	32	80	112 (40)
How often do you experience regurgitation?	Never	0	20	20 (7.12)
	Rarely	0	44	44 (15.71)
	Occasionally	116	28	144 (51.42)
	Often	0	72	72 (25.71)
How often do you experience chest pain (burning pain)?	Never	4	76	80 (28.57)
	Rarely	0	24	24 (8.57)
	Occasionally	56	64	120 (42.85)
	Often	56	0	56 (20)
How often do you experience difficulty in swallowing?	Never	44	96	140 (50)
	Rarely	56	28	84 (30)
	Occasionally	16	32	48 (17.14)
	Often	0	8	8 (2.85)

## Discussion

This study is the first to explore the relationship between lifestyle factors and GERD specifically among Pakistani patients. Our findings reveal a higher prevalence of GERD among females compared to males, a trend that contrasts with a study conducted in Korea, which found no significant association between gender and GERD symptoms [10]. This discrepancy highlights the potential influence of regional and cultural differences on GERD prevalence and underscores the need for further research to understand the factors contributing to these variations.

In our study, we observed a significant relationship between BMI and GERD, aligning with the findings of other studies that have demonstrated a similar association [11-13]. However, this contrasts with a study conducted in Korea [10], which did not find a significant relationship between BMI and GERD. These divergent results suggest that the influence of BMI on GERD may vary across different populations, possibly due to genetic, environmental, or lifestyle factors. This inconsistency underscores the necessity for further cross-cultural research to elucidate the underlying mechanisms and contributing factors that affect the BMI-GERD relationship.

GERD has a well-documented association with increased food intake, as demonstrated by a study conducted in China [14]. Our findings similarly indicate that GERD is more prevalent among individuals who eat beyond the point of fullness and consume meals a few hours before bedtime. This aligns with existing literature, reinforcing the idea that late-night eating and overconsumption are significant risk factors for GERD [14]. These behaviors likely exacerbate reflux symptoms due to increased gastric pressure and the propensity for food to reflux into the esophagus when lying down shortly after eating. Further studies are warranted to explore these dietary patterns and their impact on GERD in different populations.

Our study did not find a significant correlation between GERD and chronic gastrointestinal conditions, including Crohn's disease and inflammatory bowel disease (IBD), a finding not corroborated by additional research [15].

While GERD is often associated with alcohol consumption [16], our study presents a contrasting finding. Due to religious and cultural practices in our study population, alcohol consumption is minimal, and thus, its impact on GERD was not observed. This deviation from established associations highlights the importance of considering cultural and religious contexts when studying GERD risk factors. It suggests that other factors may play a more significant role in the development of GERD in populations where alcohol consumption is not prevalent.

Our study's findings on the impact of tomato consumption, carbonated drinks, and caffeinated beverages on GERD are consistent with those of other studies [17]. These dietary factors have been shown to exacerbate GERD symptoms due to their acidic nature and ability to relax the lower esophageal sphincter, leading to increased acid reflux. This alignment with existing research underscores the significance of dietary habits in the management of GERD and supports the need for dietary modifications as part of a comprehensive treatment approach.

Limitations

The study was conducted at a single medical center; the results may not be representative of the broader population. The study is limited to Pakistani patients, and the findings may not be generalized to populations with different cultural and dietary habits. Due to cultural and religious practices, alcohol consumption is minimal in the study population, limiting the ability to explore its impact on GERD.

Implications

The following implications can overcome limitations: 1) The confinement to a single medical center may limit the generalizability of our findings. To overcome this, future research should incorporate multi-center studies involving diverse geographic regions. This would provide a broader representation and enhance the applicability of the results to a wider population. 2) Our study's focus on Pakistani patients restricts the applicability of the findings to populations with different cultural and dietary habits. Expanding the study to include participants from various cultural backgrounds will provide a more comprehensive understanding of the relationship between dietary habits and GERD across different populations. 3) Given the minimal alcohol consumption in our study population due to cultural and religious practices, our ability to assess its impact on GERD is limited. Future studies should include populations where alcohol consumption is more prevalent. This will allow for a more thorough investigation into how alcohol intake influences GERD.

## Conclusions

Our study is the first to examine the relationship between lifestyle factors and GERD among Pakistani patients. Our findings highlight significant associations between GERD and several factors, including gender, BMI, dietary habits, and chronic gastrointestinal conditions. Notably, cultural and regional differences appear to influence GERD prevalence and its risk factors, as demonstrated by the minimal impact of alcohol consumption in our study population.

## Appendices

Questionnaire

Participant Information

Age: _____                   Gender:

Height (m): _____             Weight (kg): _____                      BMI: _____

1) How long have you been diagnosed with GERD?

a) Less than 1 year                               b) 1-2 years                         c) More than 2 years

2) Do you have any other gastrointestinal conditions (e.g., IBS, Crohn's disease)?

a) Yes                                  b) No

3) How many meals do you eat per day?

a) 1                                      b) 2                               c) 3

d) More than 3

4) How often do you consume

4a) Fried or fatty foods:

a) Never                                  b) Rarely                        c) Occasionally

d) Often

4b) Spicy foods:

a) Never                                  b) Rarely                                 c) Occasionally

d) Often

4c) Citrus fruits/juices:

a) Never                               b) Rarely                                  c) Occasionally

d) Often

4d) Tomato-based products:

a) Never                                  b) Rarely                        c) Occasionally

d) Often

4e) Chocolate:

a) Never                             b) Rarely                       c) Occasionally

d) Often

4f) Caffeinated beverages (coffee, tea, soda):

a) Never                          b) Rarely                      c) Occasionally

d) Often

4g) Alcoholic beverages:

a) Never                        b) Rarely                           c) Occasionally

d) Often

4h) Carbonated drinks:

a) Never                         b) Rarely                       c) Occasionally

d) Often

5) Do you eat large meals?

a) Never                          b) Rarely                        c) Occasionally

d) Often

6) Do you eat late at night (within 2 hours of going to bed)?

a) Never                             b) Rarely                          c) Occasionally

d) Often

7) Do you smoke?

a) Yes                                                    b) No

8) Do you exercise regularly?

a) Yes                                                             b) No

9) How often do you experience

9a) Heartburn:

a) Never                 b) Rarely                        c) Occasionally

d) Often

9b) Regurgitation:

a) Never              b) Rarely                               c) Occasionally

d) Often

9c) Chest pain:

a) Never                     b) Rarely                         c) Occasionally

d) Often

9d) Difficulty swallowing:

a) Never                         b) Rarely                                     c) Occasionally

d) Often

10) Do your symptoms affect your daily activities?

a) Yes                                                             b) No
